# CAR-T cells for Colorectal Cancer: Target-selection and strategies for improved activity and safety

**DOI:** 10.7150/jca.50509

**Published:** 2021-01-21

**Authors:** Huali Li, Chao Yang, Huangrong Cheng, Shuoyang Huang, Yongbin Zheng

**Affiliations:** Department of Gastrointestinal Surgery, Renmin Hospital of Wuhan University, Wuhan, Hubei 430060, P.R. China.

**Keywords:** colorectal cancer, chimeric antigen receptor-T (CAR-T) cell, tumor associated antigens, toxicity, strategies

## Abstract

Chimeric antigen receptor-T (CAR-T) cell immunotherapy is a novel method that is genetically engineered to recruit T cells against malignant disease. Administration of CAR-T cells has led to progress in hematological malignancies, and it has been proposed for solid tumors like colorectal cancer (CRC) for years. However, this method was not living up to expectations for the intrinsic challenges posed to CAR-T cells by solid tumors, which mainly due to the lacking of tumor-restricted antigens and adverse effects following treatment. New approaches are proposed to overcome the multiple challenges to alleviate the difficult situation of CAR-T cells in CRC, including engineering T cells with immune-activating molecules, regional administration of T cell, bispecific T cell engager, and combinatorial target-antigen recognition. In this review, we sum up the current stage of knowledge about target-selection, adverse events like on/off-tumor toxicity, the preclinical and clinical studies of CAR-T therapy, and the characteristics of strategies applied in CRC.

## Introduction

Colorectal cancer (CRC) is a major public health problem globally, according to statistics, approximately 147,950 individuals will be diagnosed and 53,200 will die with this disease in 2020 1. According to the data, CRC is the third commonly diagnosed malignant disease in males with 9% and females with 8%, the third for mortality (10.7%), and metastatic CRC (mCRC) is usually associated with poor prognosis, with 5-year relative survival rate ranges from 14% to 90%, corresponding to patients diagnosed with advanced disease and localized disease, respectively 1, 2. Multiple pieces of research have demonstrated the critical role of the immune system in controlling cancer growth, more importantly, has led the way to new strategies that promote active or passive immunotherapy against cancer 3, 4. The application of targeted immunotherapies can promote a better clinical benefit for patients with CRC. But the tumor type of most CRC patients with microsatellite instability-high (MSI-h) which has a markedly higher mutational load than microsatellite stable (MSS) is a barrier to the benefit of immunotherapy in those patients 5. Despite the immunogenicity of the subtype with a higher mutational load, CRC is prone to escape immunosurveillance for several mechanisms 6. Hence a series of strategies are recommended to rescue the therapeutic activity of the immune system, including active methods like immune checkpoint blockade, co-stimulatory signal domain, cancer vaccines 7-9, and passive approaches like adoptive cell therapy (ACT) and monoclonal antibodies 10, 11. And chimeric antigen receptor-T (CAR-T) cell therapy has demonstrated remarkable progress in cancer in the last decade.

CAR-T cell therapy is a form of cancer treatment in which the recipient's own T cells are extracted and genetically modified before reinfusion. It is an extraordinary concept proposed by Z Eshhar in 1989 12, which attempts to express functional chimeric receptors that recognize tumor antigen in a non-histocompatibility complex molecules (MHC)-restricted manner, paving the way for the design at the will of T-cell receptors of any desired specificity. As compared to the traditional cell-mediated therapies, CAR-T cells possess improved specificity and cytotoxicity from major MHC through single-chain variable fragment (scFv) in addition to the T cell receptor 13, 14. The CAR construct is composed of an extracellular scFv linked via a spacer, transmembrane domain, and intracellular signaling molecules that are capable to trigger the effector functions of T cells 15. T cells are transfected with CAR structures through mRNA, viral vector transduction, or plasmid transfection to redirect them toward pre-defined target cells 16. The CAR structure initially contained only the CD3ζ domain which is equipped to recapitulate “signal 1” of T cell activation, known as “first-generation” CAR 17; it later evolved into a more complex form in which intracellular costimulatory domains were added (CD3ζ plus 4-1BB or CD28 signaling domains), giving rise to second-generation 18; and the third-generation added 4-1BB and CD28 domains simultaneously, which have augmented T cell functions including cell proliferation and persistence 19; the latest fourth-generation CAR is with a transgenic 'payload' to shape the tumor environment (TME) by the inducible release of transgenic immune modifiers 20. Such transferred genetically-modified T cells are of unique pharmacokinetic properties, due to their ability to replicate and persist long-term, following a single administration. Furthermore, the use of synthetic immune receptors may impact signaling pathways involved in T cell function and survival in unexpected ways.

The method of adoptive transfer of CAR-T cells that takes advantage of the cellular immune system against cancer has demonstrated impressive success in hematologic malignancies in particular of CD19 positive B cell malignancies, and it has been proposed for solid tumors like CRC, targeting surface proteins such as natural-killer group 2, member D (NKG2D), the ligands of which partially upregulate in tumor cells, and carcinoembryonic antigen (CEA) 21, 22. Whereas increased studies dedicated to improving the treatment efficacy of CAR-T cells have shown encouraging progress, results concerning solid tumors are not sufficient to advance their clinical application. The development of CAR-T cell therapy in solid tumors has stagnated as a result of the lack of tumor-restricted antigens which are also called tumor specific antigens (TSAs), the hostile TME, and inadequate T cell persistence 23, 24. Continuous efforts are driving the development of basic research in this field, and there are several studies entered clinical trials (shown in **Table 1**) and a variety of combinational strategies are proposed for further improving efficacy and safety. Each strategy has its mechanism of action and has various advantages or limitations as summarized in **Table 2.**

## Targets and Toxicities

### The possible targets of CARs

Targeting truly tumor-restricted antigen is the key to successful CAR-T therapy. As expected, CAR-T cell therapy can not only eliminate tumor cells but also reach the specificity which ensures clinical safety if the targets belong to TSAs. But little is known about viable TSA targets, and the targets recognized by the CAR are always tumor associated antigens (TAAs). Here, we sum up the possible targets applied in CAR-T therapy for CRC, with corresponding expression profile, preclinical and (or) clinical studies (shown in **Table 1**).

Studies have described guanylyl cyclase2C (GUCY2C) as a possible target for CARs in mice models 25. GUCY2C is a membrane-bound cyclase and its cell surface expression is confined to the apical surfaces of intestinal epithelial cells and a subset of hypothalamic neurons 25, 26. The anti-tumor activity of GUCY2C-specific CAR-T cells has been confirmed in both human and syngeneic CRC xenograft models which used murine T cells, but CARs produced from 5F9 scFv (GUCY2C-scFv) didn't show cross-reactivity with murine GUCY2C, limiting the quantification of intestinal toxicity in mice models 25. And there has not been seen the clinical application of GUCY2C CAR-T cells in patients with CRC yet.

Epithelial cell adhesion molecule (EpCAM) is proposed to be an emerging biomarker for circulating tumor cells (CTCs) and is recognized as a novel target for adoptive T cell therapy 27. As reported, third-generation EpCAM CAR-T cells were generated to identify the specificity of EpCAM to CRC cells and models, demonstrated lytic cytotoxicity to target cells and secreted cytotoxic cytokines including tumor necrosis factor α (TNF-α) and interferon γ (IFN-γ) in an EpCAM-dependent manner. Infusion with these CAR-T cells significantly restrained tumor growth and development in xenograft mice models 28. However, EpCAM is expressed in most healthy adult tissues, mainly in the basolateral cell membrane of simple, transitional and pseudo-stratified epithelial 29.

Another NKG2D-based CAR-T cell therapy also showed cytotoxicity against CRC cells in a dose-dependent manner, significantly suppressed tumor growth and extended overall survival of mice. Furthermore, NKG2D-positive lymphocyte infiltration was found in the tumor site of NKG2D CAR-T cells-treated mice, but it was also accompanied by gradual weight loss 30. A clinical trial of NKG2D CAR-T cells targeting NKG2D ligands (NKG2DLs) has also been reported recently in which two autologous and allogeneic NKG2D CAR-T cells were investigated in mCRC patients through a 3+3 design evaluating 3 DL (1×10^8^, 3×10^8^ and 1×10^9^ cells per infusion), showed that DL-1 and DL-2 were completed without any dose-limiting toxicity occurrence 10. In humans, NKG2DLs consist of MHC class I-related chain A (MICA) and B (MICB), and six unique long 16 binding protein (ULBP1-6). These molecules are present at low or undetectable levels on normal tissues but significantly upregulated in multiple malignant tumors including CRC 30, 31.

Human epidermal growth factor receptor 2 (HER2) is an oncogene that encodes for a transmembrane glycoprotein receptor that functions as an intracellular tyrosine kinase and is a member of the HER2 (HER2/ERBB) family 32. In humans, HER2 is present in developing epithelial structures of the respiratory and digestive systems, the ependymal cells lining the ventricles of the central nervous system 33. In a recent report, HER2-targeted CAR-T cell confirmed its target potential, showed a similar line of efficacy against HER2^+^ tumors, including tumor regression or even elimination of CRC xenograft and protection of relapse, achieving improved survival benefit compared with the corresponding control group 34.

As is known, CEA is an acid glycoprotein which is the most common tumor marker in CRC currently. In a phase I trial, CAR-T cells were applied in 10 CEA positive CRC patients with escalating dose level(DL) that ranged from 1×10^5^ to 1×10^8^ (CAR^+^/kg cells). Seven patients who had experienced progressive disease during previous treatment were stable following treatment with CAR-T cells, moreover, two of them remained stable for more than 30 weeks, another two even showed tumor regression 21. But there were several adverse events observed after cell therapy, including fever, lymphocyte count decrease and duodenum perforation. It is unclear whether the general expression of CEA in normal human tissues (shown in **Table 1**) is responsible for these adverse events, but the potential safety risk does exist.

According to Hege K.M 35, tumor associated glycoprotein 72 (TAG-72) was applied as tumor targets in CAR therapy, and cells were administered using two methods, intravenous infusion and hepatic artery infusion. Detectable persistence of CAR-T cells was observed in blood, and the trafficking to tumor sites was recognized through tumor biopsy 35. Their findings demonstrated the anti-tumor efficacy of TAG-72 CAR-T cells but accompanied with the limitation that large metastatic deposits were resistant to T cells and evaded the immune attack. CAR-T cell therapy applied in patients with solid tumors has encountered many challenges due to the own nature of solid tumors and the presence of immunosuppressive immune cells, which prevent the successful application of this method in the clinic. And there is a series of ongoing trials targeting antigens like MUC1 (NCT02617134) or CD133 (NCT02541370) that are established to further assess the safety and feasibility of CAR-T cells for CRC (shown in **Table 1**).

In addition to targeting proteins exposed on tumor cell-surface, researchers also considered tumor stem cells (TSCs) and the process of epithelial-mesenchymal transition (EMT) as possible intervention targets, as both of which are involved in tumor progression 36, 37. Given the invasive nature of CRC, doublecortin-like kinase 1(DCLK1) which plays a critical role in tumor growth and regulating the process of EMT may be a novel target for immunotherapy in CRC 38. As reported, T cells engineered with DCLK1-scFv(CBT-511) induced cytotoxicity and increased IFN-γ release when co-incubated with CRC cells in two-dimensional, furthermore, higher levels of IFN-γ were observed when cells were cultured in three-dimensional 39. *In vivo*, CBT-511 CAR-T cells blocked the growth of subcutaneous xenograft tumors derived from LoVo CRC cells, but there was no further detection for histotoxicity when a dramatic release of IFN-γ was observed 39. And it is reported that DCLK1 not only upregulates in tumor cells but also scattered in the intestinal epithelium where the lower crypts in normal murine and human intestines are most frequently observed, heightening concern about toxicity 40. Although there are few TSA available or even reported in CRC, increasing designs are focused on dual TAA targeting, and even by optimizing the CAR structure and combining the density difference of target antigen to indirectly regulate the immune-related toxicity caused by lack of tumor-specific antigen.

### The off-tumor toxicity

Apart from the considerable advantage of CAR-T therapy showed in solid tumor models, the inherent divergence between mice and humans, the sharing-expression pattern of most antigens in the human body that is comparable to distribution in mice are two gaps difficult to bridge 26, 30, 48, 49. Adoptive T cell therapy has enormous potential against cancer, it is also a double-edged sword in solid tumors where non-malignant tissues suffered 'indiscriminate attacks' in some cases during treatment. The most typical resulting “on-target off-tumor” toxicity is a case report, in which a mCRC patient received 1×10^10^ third-generation CAR-T cells intravenously targeted ERBB2 (also referred to as HER2) overexpressing tumors 50. The patient suffered respiratory distress, displayed a dramatic pulmonary infiltrating within 15 minutes after cell infusion, and died 5 days after infusion despite intensive medical intervention 50. The most important reason for this case is that a large number of ERBB2 CAR-T cells localized to the lung immediately following infusion, and the synchronous recognition of ERBB2 that exposed on normal epithelial cells triggered the release of cytokines. The analogous toxicity has also been shown in a recent report, in which two advanced CRC patients experienced lymphopenia after administrated with CD133 CAR-T cells, one of them even experienced hyperbilirubinemia (direct bilirubin) with grade 3 and lasted 3 weeks 51. The simultaneous expression of CD133 in a variety of hematocytes such as hematopoietic stem cells and progenitor cells may be the main reason for hematopoietic system toxicity, and bilirubinemia toxicity may be partially put down to CD133 that was recognized as a marker of endothelial cells. It raises concern that such “on target off-target” toxicity may also occur on antigens with a similarly sharing-expression profile. For instance, TAG-72 described above is expressed in a variety of normal tissues including the normal mucosa, the endometrium during the secretory phase, and the fetal tissues 26, 52-54.

### The on-tumor toxicity

Besides the “on target off-target” toxicity summarized above which damaged both malignant and normal tissues, cytokine release syndrome(CRS) is one of the most frequently observed toxicity of CAR-T cell therapy as a result of overactivation of T cells 55, 56. CRS initially manifests with symptoms including fatigue, fever, and myalgias, which may be accompanied with hypoxia and hypotension, even can develop into life-threatening outcomes with capillary leak 57, 58. As previously reported, the levels of several cytokines like interleukin 2 (IL-2), IFN-γ and TNF-α are markedly elevated in patient's serum after treatment with CAR-T cells 59-61. The most commonly studied CAR therapy in hematological malignancies is usually with various adverse symptoms caused by such high levels of cytokine in serum. In terms of CRC, symptoms in accord with low-grade CRS were observed in both trial C-9701 (intravenous infusion) and trial C-9702 (hepatic artery infusion) when administered TAG-72 CAR-T cells 35. The most frequently observed grade 1 to 2 adverse events in trail C-9701 included chills accounted for about 70%, and dizziness, fever, paresthesia, each term was about 30%, all suggesting low-grade toxicity with IFN or CRS. Other symptoms attributed to IFN-α were nausea, malaise, flu syndrome and abdominal pain, accounting for 50%, 40%, and 30%, respectively. In trial C-9702, one patient experienced a recurrent transient syndrome of fever, increased bilirubin and anemia 2 months following the last infusion^35^. Infusion-related toxicities were common, including fever, nausea, vomiting, increased bilirubin, headache and anemia. It is recognized that grading CRS is of great benefit to guide the management of adverse events. Moreover, attempts to alleviate and even to prevent toxicities are proposed, and various safety strategies that focus on optimizing CAR structure are proposed, including combinatorial target-antigen recognition, switch-mediated CAR. Synthetic Notch receptor as a novel method has demonstrated efficacy in hematological malignancies 62, 63. All these approaches to alleviating toxicity have further promoted the treatment of CAR-T cells in malignant tumors.

## Strategies to improve safety

### Bispecific T cell engager

**Figure 1:**
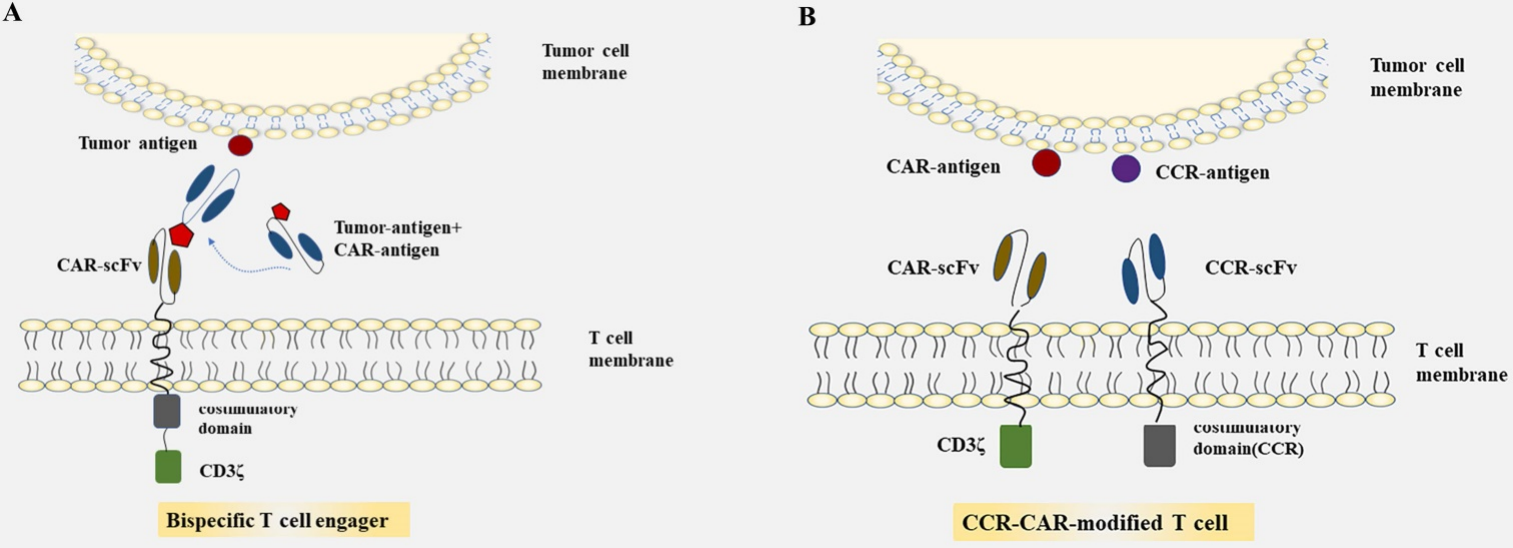
Summarized strategies for CAR-T cells to overcome toxicity in CRC. **A:** CAR-T cells were engineered to express CARs that do not directly recognize antigens on target cells, but are recruited to effector cells through bifunctional small molecule “switch”. **B:** CAR-T cell are transduced with optimized constructions include the CAR structures that provide inefficient activation signals of T cells when bind to one antigen and chimeric costimulatory receptors(CCR) recognize another antigen.

As many unfavorable factors exist between CAR-T cells and solid tumors, a series of bispecific antibodies emerged that bind to signal domain CD3ζ directly, to recruit T cells. Bispecific single-chain antibodies (bscAbs) that are referred to as “BiTE” molecules are one kind of such antibodies 64. In a preliminary study, a kind of bscAbs combining various scFv recognizing human CEA with a de-immunized scFv recognizing human CD3 were constructed for treatment against CEA positive tumors 65. Results showed that CEA/CD3-bscAbs redirected human T cells to lyse CEA^+^ tumor cells *in vitro* and *in vivo*, and the bscAbs could mediate efficient regression in different tumor cell models, though the available CEA-binding site is under sufficient. Furthermore, the cytotoxic activity of a subset of CEA/CD3-bscAbs was not competitively inhibited by soluble CEA at concentrations that exceeded levels found in the serum of most patients 65. As shown in previous reports, these bispecific molecules could transiently connect resting T cells to tumor cells, leading to activation of T cells and lysis of tumor cells 65-67. The bscAbs showed cross-reactivity with normal human tissues, however, and there is still the possibility of damaging normal tissues. Interestingly, progress has been made in the recombinant bispecific molecules. Engineering T cells with CARs that are activated by bifunctional small molecule “switch” consisting of a scFv recognizing antigen expressed on tumor cells conjugated to a protein combined with CAR-scFv, can redirect and regulate the activity of CAR-T cells toward target cells both in a dose-dependent and specific manner, and induced immune activity against cancer *in vitro* and *in vivo* 68, 69 (**Fig. 1A**). The system which used fluorescein isothiocyanate (FITC)-specific CAR-T cell and CD19- or CD22-directed bifunctional molecule showed high cytotoxicity to CD19- or CD22-positive cells 69. The activation and proliferation of T cells not only depend on the presence of tumor cells that express the target antigen, but also are strictly regulated and dose titratable by bifunctional molecules. The safety of CAR-T therapy in solid tumors may be significantly improved through such a BiTE-based method.

### Dual-CAR-modified T cell

Most of the CARs are designed with single target, which may lead to immune escape and selective survival of antigen-negative tumor cells on account of tumor heterogeneity 70-72. The therapeutic effect of single CAR treatment correlates with the own properties of the target antigen, and with its role in tumorigenesis and tumor growth. Researchers have proposed strategies to render T cells specifically in the lack of TSAs, where T cells are engineered with optimized constructions including the CAR structures that provide inefficient activation signals of T cells when binding to one antigen and chimeric costimulatory receptors (CCR) recognize another antigen 73, 74 (**Fig. 1B**). T cells expressing PSMA-CCR and suboptimal activation receptor (low-affinity PSCA-specific scFv) were effectively activated when co-cultured with PSCA^+^PSMA^+^ instead of PSCA^+^PSMA^-^ cells, and showed enhanced protection against tumor cells *in vitro* and *in vivo* 74. In a dual-receptor CAR (dCAR) study, they constructed a tandem structure consisted of CEA-CD3ζ and MSLN-4/1BB signaling domains that are physically separated, and the activation of CAR-T cells was efficiently regulated by CEA and MSLN, respectively. And dCAR-T cells exerted reinforced efficacy compared to single-receptor CAR with only one signaling pathway, under the existence of CEA^+^MSLN^+^ tumor cells 75. Nevertheless, multiple parameters of such dCAR system have to be precisely controlled for practical application, including the signaling intensity, the level and ratio relative to each synergic receptor. Moreover, the ability to truly exhibiting switch controllability of the dCAR system can also be complicated, due to the different levels of target antigen expressed on various tissues and organs in the human body.

The design of “switch” mediated CARs through small molecules that control the therapeutic effect of T cells while still retaining antigen specificity, may make it possible for these parameters controlling of CAR-T immunotherapy in solid tumors 76-78. In these physically separated receptors, antigen-binding site and intracellular signaling domain integrate into a complete signal chain only with the help of heterodimerizing small molecules, including rapamycin analog AP21967 (rapalog) that induces heterodimerization of FK506 binding protein (FKBP) domain and T2089L mutant of FKBP-rapamycin binding component (FRB*) 76, gibberellin analog containing an acetoxymethyl group (GA3-AM) induced dimerization system which consists of gibberellin insensitive dwarf1 (GID1) and gibberellin insensitive (GAI) 78, and rimiducid (AP1903) induced inducible MyD88/CD40 (iMC) and antigen-CD3ζ signaling domain 77. This titratable pharmacologic regulation can not only combine with dual antigens, or even multiple antigens, but make it possible for precisely controlling the intensity and timing of T cell activity, and even the location of T cells, thereby improving antigen-specificity and dose-dependent safety, if this dimerization system cooperates with the dCAR system described above.

## Strategies to enhance efficacy

Although studies have demonstrated promising prospect of CAR-T therapy in some mice models, the full and persistent function of CAR-T cells has been limited for some unique challenges posed by solid tumors: (a) the chemokine/chemokine receptor mismatches, antigen loss or heterogeneity; (b) the barriers characterized by physical and chemical nature of the TME: high tissue pressure, hypoxia, abnormal vasculature, and unfavorable immune cells; (c) the inherent inhibitory mechanisms of T cells 79, 80. Since then, attempts to strengthen the functions of CAR-T cells in preclinical models and to further prolong the overall survival of CRC patients emerged.

### Engineered T cell with immune-activating molecules

Cytokines that play a crucial part in T cell activation, proliferation, and cytotoxicity are one of the best-characterized molecules and delivered by CAR-T cells 81, 82. Preclinical studies have demonstrated that the involvement of cytokines like IL-2, IL-7, IL-12, and IL-21 enhanced the immune activity of the immune system, and may reverse the inhibitory TME to an immune stimulatory environment 83-86. Of these immune-activating molecules, IL‐12 is a central one to assist in cancer treatment 87. In Chi's group, they confirmed that combination use of CEA CAR‐T cells and recombinant human IL-12 (the dose of IL-12 were 1, 10, 50, 100, 200, 500, and 1000 U/mL, respectively) significantly enhanced anti-tumor efficacy *in vivo*, showed improved T cell efficacy and increased level of serum cytokines than CEA CAR‐T cells alone. The cytotoxicity of CAR-T cells improved with the increase of IL-12 dose within 50 U/mL and is close to saturation when IL-12 dose is more than 50 U/mL 87. Another kind of “two in one” approach in which researchers engineered mesenchymal stem cells (MSCs) to release both IL-7 and IL-12 for the tremendous plasticity of MSCs and their capability to secrete a mass of immune-activating molecules, also showed a mutual interaction between interleukins and CAR-T cells 86, 88-90. The improved CAR-T cell activation through modified MSCs was judged by cytokine production and cytolytic activities, and the enhanced elimination of tumor cells in a xenograft mouse model of CRC was confirmed. The safety and feasibility of EGFR and EGFR IL-12 CAR-T cells in the treatment of mCRC are evaluated currently in phase I and II trials (NCT03542799, NCT02959151). Other interleukins combined with CAR-T cell include IL-18, IL-23, IL-15, which have yet been seen in CRC 91-93. Such a combinatory strategy has great potential to boost CAR-T cells for stronger and safer efficacy, but the significant increase of serum cytokines levels resulted from enhanced T cell activation was also observed 87, 94.

**Figure 2:**
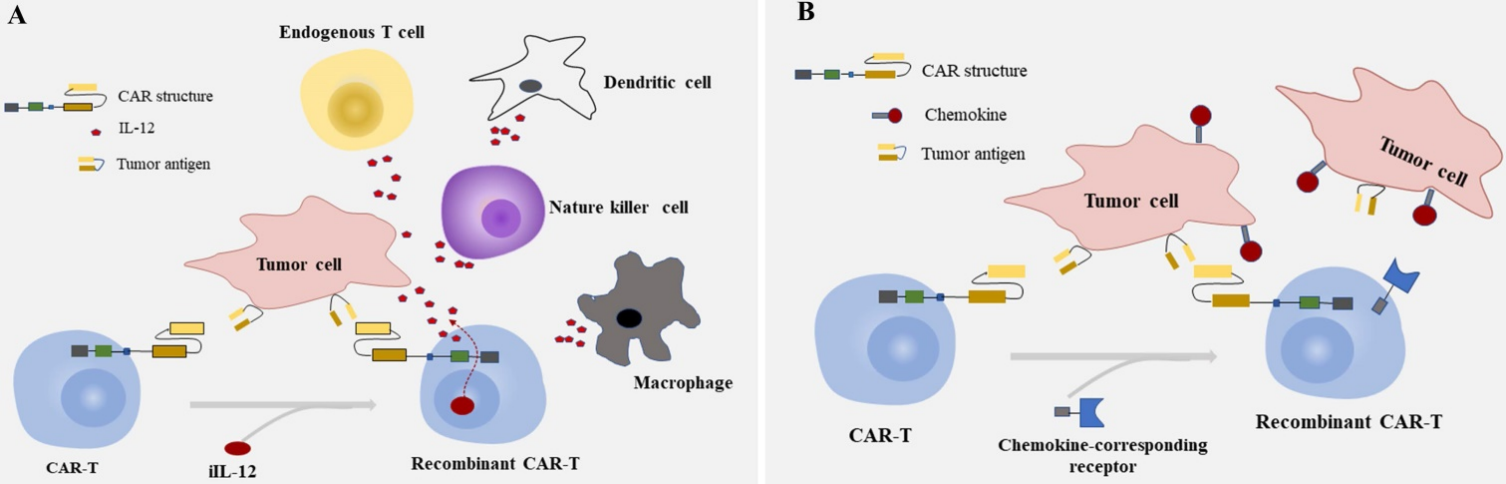
Summarized strategies for CAR-T cells to enhance efficacy in CRC. **A:** Engineering T cells to secrete inducible IL-12(iIL-12) which are driven by the CAR in tumor site. The interleukins delivered by CAR-T cells deposit in the tumor lesion could attract innate immune cells, like NK cells ,endogenous T cells, dendritic cells and macrophages modulating the tumor microinvironment. **B:** Furnishing CAR-T cells with genes of chemokine-corresponding receptors to chemokines secreted by solid tumors improves the homing of T cells and the capability of migration and achieving access to tumor site.

As is known, IL-12 contributes to cell differentiation of Th1 and proliferation of natural killer (NK) cell, while IL-2 and IL-21 work co-operatively to enhance the cytolytic activity of CD8^+^T cells and NK cells, induce a sustainable CD8^+^ response 95. Engineering T cells to secrete these molecules which are driven by the CAR in tumor site to avoid the systematical increase of cytokines would be desirable 96, 97. The interleukins delivered by CAR-T cells deposit in tumor site could attract innate immune cells like macrophages and NK cells attacking tumor cells that escape from CAR-T cells due to the lack of targets recognized by CAR 98 (**Fig. 2A**). Some bench studies also showed that CAR-T cells genetically engineered to constitutively secrete cytokines can reverse the unfavorable TME not only by enhancing the proliferation and expansion of T cells but also by activating the immune effector cells 82, 98, 99.

Chemokine and chemokine receptor mismatch is considered to be one of the key factors that limit T cell location in tumor deposits, as active trafficking of T cells to tumor sites in part be attributable to the correct match between chemokine receptors on T cells and chemokines exposed on tumor cells 80, 100, 101. To overcome the above factor impeding the migration of CAR-T cells to tumor mass, furnishing CAR-T cells with chemokine (C-C-motif) receptor (CCR), C-X-C motif chemokine receptor (CXCR), or other receptors to chemokines secreted by tumor cells is another option to achieve effective homing of CAR-T cells and improve the capability of migration 102-104 (**Fig. 2B**). As shown in recent research, CXCR1/CXCR2 modified CARs significantly improved T cell persistence and migration, induced durable response, and long-lasting immunologic memory in preclinical models of aggressive tumors 105. The identical cytotoxicity was also observed in a case of hepatocellular carcinoma (HCC), in which the migration ability of T cells engineered with CXCR2 significantly enhanced *in vitro*, showed accelerated cell trafficking and tumor-specific accumulation *in vivo* 102. Given the characteristics between T cells and tumors, it would be of great prospects for engineering T cells with CXCR modified CARs in solid tumors like CRC. But caution should be raised as some preliminary data indicate that the continuous secretion of high levels of intracellular calcium resulted from chronic stimulation of T cells through chemokine receptors may induce T cell hypofunction 106, 107.

### Regional administration of T cell

An opportunity to avoid the traffic load in solid tumors might be regional administration of T cell, which has made modest success in some cancer 108-110. In CRC with liver metastases, Burga has demonstrated that cell delivery to liver metastases was optimized through regional intrahepatic infusion 111. Furthermore, the efficacy of CAR-T cells could be enhanced when mice received cells in tandem with blockage of inhibitory immune molecules, including myeloid-derived suppressor cells (MDSC) depletion, GM-CSF neutralization that are to prevent MDSC expansion, and PD-L1 inhibition. They later carried out a preclinical study about intraperitoneal (IP) delivery of CEA CAR-T cells against peritoneal carcinomatosis on the basis above, showed superior anti-tumor efficacy against CEA^+^ peritoneal tumors, prolonged protection against tumor re-challenges, and effector memory T (T_cm_) cells increased over time when compared with systemic infusion 112. Moreover, in combination with depleting antibodies for MDSC and regulatory T cells, IP delivery of CAR-T cells further protected mice from peritoneal deposits. Data also showed that IP delivery of T cell did lead to high levels of systemic IFN-γ following treatment. Regional administration of T cells can indeed improve the situation of inadequate trafficking and persistence of T cell, and the blockage of inhibitory molecules can further reverse the unfavorable TME, rescue the efficacy of T cell 113, 114. Whether patients with CRC can generally benefit from strategies combined with immune checkpoint blockage remains to be confirmed in further studies.

## Conclusions and Further perspectives

In light of the recent progress of CAR therapy, especially in hematological malignancies, a series of optimization strategies for more specific and safer benefits in solid tumors are in need even with lots of limitations to this method existed. As summarized in this review, there are various targets to choose from for the CAR treatment in CRC and many promising therapeutic strategies have been proposed and shown success in preclinical models. The important role of CAR therapy in solid tumors is confirmed in a variety of studies. As discussed above, an ideal target candidate for the CAR structure should meet the conditions including significantly up-regulated in tumor cells and at low or even undetectable levels on normal tissues. But it is usually hard to find out. We hold the opinion that it is a promising way to combine the dCAR system with heterodimerizing small molecules, to binding multiple antigens, thus the activation of T cells can be controlled by adjusting the density of antigens to achieve accurate targeting of tumor cells, under the control of small molecules. When clinicians hold the controllable “switch”, these impressive results confirmed by *in vivo* and *in vitro* studies may be “replicated” to patients with solid tumors more safely.

Besides engineered with T cells, NK cells are proposed to be another vector of the CAR structure and considered to be less prone to cause graft-versus-host disease (GVHD), in addition, the CAR-NK cells may expand the horizon of treatment in solid tumors and extend to allogenic CAR therapies 115, 116. Personalized modification of CAR-T cells, such as integrating with anti-tumor cytokines, changing cell infusion path, and a series of novel CAR structure-design are some assumed changes that can make a better prospect. Moreover, engineering T cells with a more suitable subset, manipulating immunosuppressive checkpoints such as PD1 or CTLA4, and incorporating soluble immune-unfavorable cytokines like TGF-β may also achieve durable response in solid tumors 117-119. These strategies have opened the field for further development of adoptive cell immunotherapy, and extensive works are needed for implementing these methods in CRC. As for CRC, there are varieties of researches combined PD-1 antibodies that have been identified as a possible target for immunotherapy in MSI-h CRC patients, or PD-1 disruption like CRISPR/Cas9 ribonucleoprotein-mediated editing to break up the PD-1 gene which is located in human primary T cells 120-122. Furthermore, the introduction of PD-1-specific scFvs has generated CAR-T cells with improved safety and efficacy against poorly responding tumor cells for its unique ability to reactivate the host's anti-tumor immunity and protected T cells from immunosuppression via disrupting the binding to its ligand 118. Engineering CAR-T cells to co-express a PD-1 decoy receptor that replaces the PD-1 transmembrane and intracellular signaling domains with the costimulatory domain of CD28 or IL-7 receptor is proved to show superior and persistent antitumor activity against various solid tumors, converting or competing possible inhibitory signal to improve T cell function 123, 124. And the superior CAR-T cells showed a significantly greater number of CD8^+^CAR-T_E_/_EM_ and CD8^+^CAR-T_CM_ cells. Given these favorable mechanisms, the poor efficacy of method aiming at immune checkpoint blocking alone in MSS or mismatch repair proficient (pMMR) patients with CRC may be rescued by combination with CAR-T cell therapy.

Adoptive T cell immunotherapy is a method that reactivates the immune system against cancer and has been in development for decades. The progress and experience obtained from hematological malignancies with this method have strengthened the impression of immunotherapy in cancer and make it possible for treatment in solid tumors. In this review, we have summarized CAR-T cells as monotherapy or in combination with other methods currently applied in CRC, and it is believed to bring substantial clinical benefit to CRC patients with prolonged endeavor.

## Authors' Contributions

Huali Li conceived and drafted the initial manuscript. Chao Yang, Huangrong Cheng, Shuoyang Huang taken charge of Literature searching and classification. Yongbin Zheng made substantial contributions to manuscript modifications. All authors read and approved the final manuscript for publication.

## Figures and Tables

**Table 1:** The antigens of CAR-T cells entered clinical trials in CRC

Antigen	Expression profile (normal)	Identifier	Phase stage	Type of cancer	Study start	Sponsor	Status
CEA	Epithelial of colon, tongue, esophagus and cervix, prostate; mucous cells of neck; secretory epithelia and duct cells of sweat glands; bronchial mucus 41, 42	NCT02349724	I	Colorectal, lung, gastric, breast and pancreas cancer	December 2014	Southwest Hospital	Unknown
		NCT03682744	I	Colorectal, peritoneal, gastric, breast and pancreas cancer, peritoneal metastases	September 2018	Sorrento Therapeutics	Active, not recruiting
EGFR	The majority of normal epithelial tissues (epidermal cells, fibroblasts, liver, kidney, ovary, testis, prostate, and uterus) 43, 44	NCT03542799	I	Metastatic CRC	May 2018	ShenZhen Second People's Hospital	Not yet recruiting
		NCT03152435	I/II	CRC	June 2017	Shenzhen Second People's Hospital	Unknown
MUC1	The apical surface of most epithelial cells (glandular epithelia, lactating mammary gland, many simple epithelial cells lining glands or ducts) 45, 46	NCT02617134	I/II	CRC; malignant glioma of brain; gastric carcinoma	November 2015	Hefei Binhu Hospital	Unknown
NKG2DL	Presenting at low or undetectable levels in normal human tissues 30, 31	NCT03310008	I	Colon Cancer Liver Metastasis	August 2017	Celyad (formerly named Cardio3 Bio-Sciences)	Active, not recruiting
		NCT03692429	I	CRC	November 2018	Celyad (formerly named Cardio3 Bio-Sciences)	Recruiting
HER2	Developing epithelial structures of the respiratory and digestive systems, the ependymal cells lining the ventricles of the central nervous system 33	NCT02713984	I/II	CRC, breast, ovarian, lung, gastric, glioma and pancreatic cancer	March 2016	Southwest Hospital of Third Millitary Medical University	Withdraw
		NCT03740256	I	CRC, bladder, lung, breast, gastric, pancretic and esophageal cancer, HNSC, cancer of the salivary gland	June 2020	Baylor College of Medicine	Not yet recruiting
CD133	Hematopoietic stem cells, CD34^+^ progenitor cells; kidney, nervous system, and epithelial tissues of the prostate 47	NCT02541370	I/II	CRC, liver, pancreatic, brain, breast, ovarian and acute myeloid and lymphoid leukemias	June 2015	Chinese PLA General Hospital	Completed

**Table 2:** The advantages and limitations of various strategies

Strategy		Advantages	Limitations
Combination with immune-activating molecules	Cytokine	Improve the activation, proliferation, and cytotoxicity of T cell	Risk of toxicity caused by high level of serum cytokines
	Chemokine receptor	Improve the capability of migration, effective homing of T cell and achieving access to tumor cells	Chronic stimulation of T cell may lead to high levels of intracellular calcium resulting T cell hypofunction
Infusion method	Regional administration	Avoid the traffic load of T cells, reduced engineered T cell depletion	Without a focus on toxicity caused by the high level of systemic cytokines like IFN-γ following infusion
Switch-mediated CAR	Bispecific T cell engager	Redirect and regulate T cell activity toward target cells in a dose-dependent and specific manner	Few available TSAs
dCAR system	CCR-CAR-modified T cell	Improved safety and enhanced specificity for combining with dual antigens	The system must be precisely controlled, including the signaling intensity, the level and ratio relative to each synergic receptor

## Abbreviations

CEA: carcinoembryonic
EGFR: epidermal growth factor receptor
MUC1: transmembrane mucins
EpCAM: epithelial cell and adhesion molecule
NKG2DL: natural killer group 2, member D ligand
HER2: human epidermal growth factor receptor 2
IFN-γ: interferon γ
dCAR: dual chimeric antigen receptor
TSAs: tumor specific antigens
CCR: chimeric costimulatory receptor
